# 3,4-*O*-(2,3-Dimethoxy­butane-2,3-di­yl)-*S*-(4-methyl­phen­yl)-1-thia-α-d-manno­pyran­oside

**DOI:** 10.1107/S1600536808018874

**Published:** 2008-06-28

**Authors:** Fei-Fei Xu, Dong Han, Lin-Na Wang, Xiang-Bao Meng, Zhong-Jun Li

**Affiliations:** a Department of Chemical Biology, School of Pharmaceutical Sciences, Peking University, Beijing 100083, People’s Republic of China; b State Key Laboratory of Natural and Biomimetic Drugs, Peking University, Beijing 100083, People’s Republic of China

## Abstract

In the title mol­ecule, C_19_H_28_O_7_S, the six-membered manno­pyran­oside and dioxane rings both display typical chair conformations. In the crystal structure, the hydr­oxy groups are involved in inter­molecular hydrogen bonds, which link the mol­ecules into chains extended along the *b* axis.

## Related literature

For details of the synthesis, see Crich *et al.* (2000 ▶).
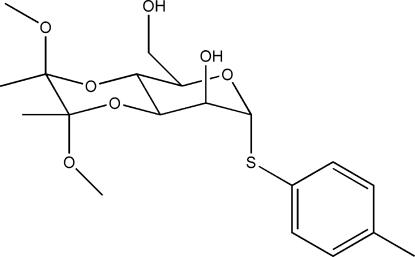


## Experimental

### 

#### Crystal data

C_19_H_28_O_7_S
*M* *_r_* = 400.47
Monoclinic, 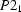

*a* = 9.8272 (6) Å
*b* = 10.3152 (6) Å
*c* = 10.2585 (6) Å
β = 100.452 (3)°
*V* = 1022.64 (10) Å^3^
*Z* = 2
Mo *K*α radiation
μ = 0.20 mm^−1^
*T* = 113 (2) K
0.32 × 0.26 × 0.18 mm

#### Data collection

Rigaku Saturn diffractometer
Absorption correction: multi-scan (*CrystalClear*; Rigaku/MSC, 2005 ▶) *T*_min_ = 0.940, *T*_max_ = 0.966
11384 measured reflections
4843 independent reflections
4168 reflections with *I* > 2σ(*I*)
*R*_int_ = 0.036

#### Refinement

*R*[*F* ^2^ > 2σ(*F* ^2^)] = 0.034
*wR*(*F* ^2^) = 0.099
*S* = 1.08
4843 reflections
257 parameters
1 restraint
H atoms treated by a mixture of independent and constrained refinement
Δρ_max_ = 0.26 e Å^−3^
Δρ_min_ = −0.22 e Å^−3^
Absolute structure: Flack (1983 ▶), 2274 Friedel pairs
Flack parameter: 0.00 (9)

### 

Data collection: *CrystalClear* (Rigaku/MSC, 2005 ▶); cell refinement: *CrystalClear*; data reduction: *CrystalClear*; program(s) used to solve structure: *SHELXS97* (Sheldrick, 2008 ▶); program(s) used to refine structure: *SHELXL97* (Sheldrick, 2008 ▶); molecular graphics: *SHELXTL* (Sheldrick, 2008 ▶); software used to prepare material for publication: *CrystalStructure* (Rigaku/MSC, 2005 ▶).

## Supplementary Material

Crystal structure: contains datablocks I, global. DOI: 10.1107/S1600536808018874/cv2406sup1.cif

Structure factors: contains datablocks I. DOI: 10.1107/S1600536808018874/cv2406Isup2.hkl

Additional supplementary materials:  crystallographic information; 3D view; checkCIF report

## Figures and Tables

**Table 1:** Hydrogen-bond geometry (Å, °)

*D*—H⋯*A*	*D*—H	H⋯*A*	*D*⋯*A*	*D*—H⋯*A*
O2—H2⋯O5^i^	0.86 (3)	1.96 (3)	2.788 (2)	161 (2)
O5—H5⋯O7^ii^	0.80 (2)	2.21 (3)	2.971 (2)	158 (3)

Symmetry codes: (i) 
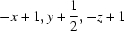
; (ii) 
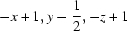
.

**Fig. 1.**
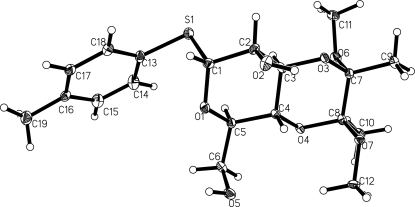
A view of (I) showing the atom-labelling scheme. Displacement ellipsoids are drawn at the 30% probability level. H atoms are represented by circles of arbitrary size.

## supplementary crystallographic information

### Comment

The skeleton of the title compound, (I) (Figure 1), is a derivative of mannopyranoside and consists of a benzene ring and a bridged ring. Both of the six-membered mannopyranoside ring and the dioxane ring display a typical chair conformation. The two methoxy groups lie in axial bonds of the dioxane ring. The hydroxy groups are involved in the intermolecular hydrogen bonds (Table 1), which link the molecules into chains extended along *b* axis.

### Experimental

The title compound was synthesized according to the known procedure (Crich *et al.*,  2000). It was obtained from 4-methylphenyl-1-thio-alpha-*D*-mannopyranoside, and then dissolved in dry methanol followed by addition of butane-2,3-dione, HC(OMe)_3_, and camphor-10-sulfonic acid. The reaction mixture was then heated to reflux for 72 h before it was cooled to room temperature and quenched by addition of Et_3_N. The reaction mixture was concentrated under vacuum, after column chromatography(hexane-ethyl acetate 1:1) yield 78% as a white solid. The compound was crystallized from hexane-ethyl acetate(1:1) to yield colourless block-like crystals after a week at room temperature.

### Refinement

The hydroxy H atoms H2 and H5 were located in a difference map and refined with distance restraints of O—H = 0.86 (3) and 0.80 (2) Å, respectively, using a riding approximation, with *U*_iso_(H) = 1.5*U*_eq_(O). C-bound H atoms were positioned geometrically (C—H = 0.95–1.00 Å) and refined as riding, with *U*_iso_(H)=1.2–1.5*U*_eq_(C).

### Crystal data

**Table:**

C_19_H_28_O_7_S	*F*(000) = 428
*M**_r_* = 400.47	*D*_x_ = 1.301 Mg m^−^^3^
Monoclinic, *P*2_1_	Mo *K*α radiation, λ = 0.71070 Å
*a* = 9.8272 (6) Å	Cell parameters from 3904 reflections
*b* = 10.3152 (6) Å	θ = 2.0–29.1°
*c* = 10.2585 (6) Å	µ = 0.20 mm^−^^1^
β = 100.452 (3)°	*T* = 113 K
*V* = 1022.64 (10) Å^3^	Block, colourless
*Z* = 2	0.32 × 0.26 × 0.18 mm

### Data collection

**Table:**

Rigaku Saturn diffractometer	4843 independent reflections
Radiation source: rotating anode	4168 reflections with *I* > 2σ(*I*)
confocal	*R*_int_ = 0.037
Detector resolution: 14.63 pixels mm^-1^	θ_max_ = 27.9°, θ_min_ = 2.0°
ω scans	*h* = −12→12
Absorption correction: multi-scan (*CrystalClear*; Rigaku/MSC, 2005)	*k* = −13→13
*T*_min_ = 0.940, *T*_max_ = 0.966	*l* = −13→13
11384 measured reflections	

### Refinement

**Table:**

Refinement on *F*^2^	Hydrogen site location: inferred from neighbouring sites
Least-squares matrix: full	H atoms treated by a mixture of independent and constrained refinement
*R*[*F*^2^ > 2σ(*F*^2^)] = 0.034	*w* = 1/[σ^2^(*F*_o_^2^) + (0.0477*P*)^2^] where *P* = (*F*_o_^2^ + 2*F*_c_^2^)/3
*wR*(*F*^2^) = 0.099	(Δ/σ)_max_ = 0.001
*S* = 1.08	Δρ_max_ = 0.26 e Å^−^^3^
4843 reflections	Δρ_min_ = −0.22 e Å^−^^3^
257 parameters	Extinction correction: *SHELXL97* (Sheldrick, 2008), Fc^*^=kFc[1+0.001xFc^2^λ^3^/sin(2θ)]^-1/4^
1 restraint	Extinction coefficient: 0.049 (3)
Primary atom site location: structure-invariant direct methods	Absolute structure: Flack (1983), 2274 Friedel pairs
Secondary atom site location: difference Fourier map	Flack parameter: 0.00 (9)

### Special details

**Table:**

Geometry. All e.s.d.'s (except the e.s.d. in the dihedral angle between two l.s. planes) are estimated using the full covariance matrix. The cell e.s.d.'s are taken into account individually in the estimation of e.s.d.'s in distances, angles and torsion angles; correlations between e.s.d.'s in cell parameters are only used when they are defined by crystal symmetry. An approximate (isotropic) treatment of cell e.s.d.'s is used for estimating e.s.d.'s involving l.s. planes.
Refinement. Refinement of *F*^2^ against ALL reflections. The weighted *R*-factor *wR* and goodness of fit *S* are based on *F*^2^, conventional *R*-factors *R* are based on *F*, with *F* set to zero for negative *F*^2^. The threshold expression of *F*^2^ > σ(*F*^2^) is used only for calculating *R*-factors(gt) *etc*. and is not relevant to the choice of reflections for refinement. *R*-factors based on *F*^2^ are statistically about twice as large as those based on *F*, and *R*- factors based on ALL data will be even larger.

### Fractional atomic coordinates and isotropic or equivalent isotropic displacement parameters (Å^2^)

**Table:**

	*x*	*y*	*z*	*U*_iso_*/*U*_eq_	
S1	0.08382 (5)	0.30225 (6)	0.13524 (5)	0.02495 (15)	
O1	0.27226 (13)	0.29547 (15)	0.36556 (13)	0.0207 (3)	
O2	0.23894 (16)	0.57684 (16)	0.39230 (16)	0.0263 (4)	
H2	0.307 (3)	0.630 (3)	0.399 (2)	0.039*	
O3	0.40302 (14)	0.62650 (13)	0.19735 (14)	0.0184 (3)	
O4	0.57553 (14)	0.40759 (13)	0.24002 (13)	0.0168 (3)	
O5	0.53143 (15)	0.23535 (16)	0.52689 (15)	0.0250 (4)	
H5	0.469 (3)	0.217 (3)	0.564 (3)	0.038*	
O6	0.49654 (18)	0.56405 (14)	0.01322 (15)	0.0219 (3)	
O7	0.67376 (13)	0.59510 (13)	0.34562 (13)	0.0174 (3)	
C1	0.1677 (2)	0.3749 (2)	0.2940 (2)	0.0210 (5)	
H1	0.0945	0.3842	0.3495	0.025*	
C2	0.2176 (2)	0.5111 (2)	0.2695 (2)	0.0202 (5)	
H2A	0.1446	0.5571	0.2054	0.024*	
C3	0.3473 (2)	0.5000 (2)	0.2110 (2)	0.0178 (4)	
H3	0.3236	0.4589	0.1214	0.021*	
C4	0.4535 (2)	0.4171 (2)	0.2984 (2)	0.0159 (4)	
H4	0.4773	0.4565	0.3888	0.019*	
C5	0.39550 (19)	0.2825 (2)	0.3079 (2)	0.0176 (5)	
H5A	0.3697	0.2451	0.2169	0.021*	
C6	0.4911 (2)	0.1905 (2)	0.3936 (2)	0.0216 (5)	
H6A	0.4447	0.1055	0.3946	0.026*	
H6B	0.5751	0.1777	0.3544	0.026*	
C7	0.5249 (2)	0.6230 (2)	0.1397 (2)	0.0170 (4)	
C8	0.6343 (2)	0.5312 (2)	0.2214 (2)	0.0171 (4)	
C9	0.5733 (2)	0.7621 (2)	0.1354 (2)	0.0247 (5)	
H9A	0.6553	0.7655	0.0936	0.037*	
H9B	0.5966	0.7962	0.2259	0.037*	
H9C	0.4994	0.8147	0.0841	0.037*	
C10	0.7551 (2)	0.5036 (2)	0.1524 (2)	0.0228 (5)	
H10A	0.8003	0.5852	0.1366	0.034*	
H10B	0.7214	0.4604	0.0676	0.034*	
H10C	0.8216	0.4471	0.2085	0.034*	
C11	0.3820 (2)	0.6183 (2)	−0.0772 (2)	0.0283 (5)	
H11A	0.3006	0.6218	−0.0345	0.042*	
H11B	0.3616	0.5640	−0.1567	0.042*	
H11C	0.4056	0.7060	−0.1021	0.042*	
C12	0.7672 (2)	0.5241 (2)	0.4431 (2)	0.0219 (5)	
H12A	0.7261	0.4401	0.4583	0.033*	
H12B	0.7849	0.5734	0.5262	0.033*	
H12C	0.8544	0.5103	0.4117	0.033*	
C13	0.0406 (2)	0.1504 (2)	0.1985 (2)	0.0225 (5)	
C14	0.1138 (3)	0.0401 (2)	0.1754 (2)	0.0304 (6)	
H14	0.1843	0.0459	0.1233	0.036*	
C15	0.0847 (2)	−0.0786 (2)	0.2279 (2)	0.0285 (5)	
H15	0.1343	−0.1537	0.2100	0.034*	
C16	−0.0166 (2)	−0.0890 (2)	0.3067 (2)	0.0235 (5)	
C17	−0.0895 (2)	0.0221 (2)	0.3287 (2)	0.0245 (5)	
H17	−0.1595	0.0165	0.3813	0.029*	
C18	−0.0619 (2)	0.1405 (2)	0.2757 (2)	0.0248 (5)	
H18	−0.1129	0.2152	0.2920	0.030*	
C19	−0.0438 (2)	−0.2154 (3)	0.3688 (2)	0.0326 (6)	
H19A	0.0348	−0.2372	0.4387	0.049*	
H19B	−0.0562	−0.2837	0.3012	0.049*	
H19C	−0.1279	−0.2082	0.4072	0.049*	

### Atomic displacement parameters (Å^2^)

**Table:**

	*U*^11^	*U*^22^	*U*^33^	*U*^12^	*U*^13^	*U*^23^
S1	0.0249 (3)	0.0269 (3)	0.0218 (3)	−0.0069 (3)	0.0010 (2)	0.0037 (2)
O1	0.0215 (7)	0.0222 (8)	0.0197 (7)	−0.0031 (7)	0.0067 (6)	0.0037 (7)
O2	0.0241 (8)	0.0287 (9)	0.0280 (9)	−0.0042 (7)	0.0097 (7)	−0.0079 (7)
O3	0.0187 (7)	0.0136 (7)	0.0234 (8)	−0.0001 (6)	0.0054 (6)	0.0032 (6)
O4	0.0210 (7)	0.0130 (8)	0.0181 (7)	0.0012 (6)	0.0077 (6)	0.0016 (6)
O5	0.0264 (8)	0.0287 (9)	0.0206 (9)	0.0015 (8)	0.0061 (6)	0.0077 (7)
O6	0.0303 (7)	0.0225 (8)	0.0127 (7)	−0.0012 (6)	0.0031 (6)	0.0015 (6)
O7	0.0200 (7)	0.0169 (8)	0.0141 (7)	0.0003 (6)	−0.0003 (6)	0.0005 (6)
C1	0.0201 (11)	0.0248 (12)	0.0173 (11)	0.0005 (9)	0.0016 (9)	0.0040 (9)
C2	0.0136 (10)	0.0226 (12)	0.0243 (11)	−0.0007 (9)	0.0027 (8)	−0.0011 (9)
C3	0.0214 (11)	0.0125 (10)	0.0209 (11)	0.0009 (9)	0.0075 (9)	0.0034 (8)
C4	0.0185 (10)	0.0156 (11)	0.0143 (10)	0.0007 (9)	0.0046 (8)	0.0014 (8)
C5	0.0188 (10)	0.0176 (12)	0.0166 (10)	−0.0023 (9)	0.0035 (8)	0.0006 (8)
C6	0.0298 (12)	0.0160 (11)	0.0201 (11)	0.0004 (9)	0.0075 (9)	0.0034 (9)
C7	0.0183 (10)	0.0165 (11)	0.0162 (10)	−0.0020 (9)	0.0033 (8)	0.0001 (8)
C8	0.0186 (10)	0.0156 (11)	0.0181 (11)	−0.0012 (9)	0.0063 (9)	0.0009 (9)
C9	0.0268 (11)	0.0184 (12)	0.0291 (12)	−0.0039 (9)	0.0055 (10)	0.0032 (9)
C10	0.0253 (12)	0.0222 (12)	0.0228 (12)	0.0027 (10)	0.0098 (9)	0.0039 (9)
C11	0.0338 (13)	0.0320 (14)	0.0164 (11)	−0.0007 (11)	−0.0027 (10)	0.0086 (10)
C12	0.0245 (11)	0.0217 (12)	0.0170 (11)	0.0041 (10)	−0.0032 (9)	0.0030 (9)
C13	0.0238 (11)	0.0243 (13)	0.0188 (12)	−0.0079 (10)	0.0023 (9)	−0.0004 (9)
C14	0.0384 (14)	0.0312 (14)	0.0251 (13)	−0.0071 (11)	0.0152 (11)	−0.0063 (10)
C15	0.0345 (13)	0.0238 (13)	0.0278 (13)	−0.0040 (11)	0.0078 (10)	−0.0108 (10)
C16	0.0205 (11)	0.0266 (13)	0.0210 (12)	−0.0074 (10)	−0.0029 (9)	−0.0021 (10)
C17	0.0206 (11)	0.0311 (13)	0.0208 (12)	−0.0040 (11)	0.0009 (9)	0.0033 (10)
C18	0.0206 (11)	0.0274 (13)	0.0261 (12)	−0.0037 (10)	0.0037 (9)	−0.0022 (10)
C19	0.0327 (13)	0.0305 (15)	0.0342 (14)	−0.0099 (11)	0.0050 (11)	−0.0009 (11)

### Geometric parameters (Å, °)

**Table:**

S1—C13	1.776 (2)	C7—C8	1.558 (3)
S1—C1	1.845 (2)	C8—C10	1.515 (3)
O1—C1	1.411 (2)	C9—H9A	0.9800
O1—C5	1.447 (2)	C9—H9B	0.9800
O2—C2	1.412 (3)	C9—H9C	0.9800
O2—H2	0.86 (3)	C10—H10A	0.9800
O3—C7	1.429 (2)	C10—H10B	0.9800
O3—C3	1.432 (2)	C10—H10C	0.9800
O4—C8	1.427 (2)	C11—H11A	0.9800
O4—C4	1.438 (2)	C11—H11B	0.9800
O5—C6	1.429 (3)	C11—H11C	0.9800
O5—H5	0.80 (2)	C12—H12A	0.9800
O6—C7	1.414 (2)	C12—H12B	0.9800
O6—C11	1.436 (3)	C12—H12C	0.9800
O7—C8	1.425 (2)	C13—C14	1.390 (3)
O7—C12	1.430 (2)	C13—C18	1.394 (3)
C1—C2	1.525 (3)	C14—C15	1.388 (3)
C1—H1	1.0000	C14—H14	0.9500
C2—C3	1.509 (3)	C15—C16	1.396 (3)
C2—H2A	1.0000	C15—H15	0.9500
C3—C4	1.511 (3)	C16—C17	1.391 (3)
C3—H3	1.0000	C16—C19	1.497 (3)
C4—C5	1.510 (3)	C17—C18	1.384 (3)
C4—H4	1.0000	C17—H17	0.9500
C5—C6	1.502 (3)	C18—H18	0.9500
C5—H5A	1.0000	C19—H19A	0.9800
C6—H6A	0.9900	C19—H19B	0.9800
C6—H6B	0.9900	C19—H19C	0.9800
C7—C9	1.515 (3)		
			
C13—S1—C1	97.68 (10)	O4—C8—C10	105.36 (17)
C1—O1—C5	115.17 (15)	O7—C8—C7	104.43 (15)
C2—O2—H2	111.3 (17)	O4—C8—C7	111.04 (16)
C7—O3—C3	112.44 (15)	C10—C8—C7	112.69 (16)
C8—O4—C4	112.53 (15)	C7—C9—H9A	109.5
C6—O5—H5	106.2 (19)	C7—C9—H9B	109.5
C7—O6—C11	115.22 (17)	H9A—C9—H9B	109.5
C8—O7—C12	115.14 (16)	C7—C9—H9C	109.5
O1—C1—C2	113.28 (17)	H9A—C9—H9C	109.5
O1—C1—S1	112.97 (15)	H9B—C9—H9C	109.5
C2—C1—S1	109.54 (15)	C8—C10—H10A	109.5
O1—C1—H1	106.9	C8—C10—H10B	109.5
C2—C1—H1	106.9	H10A—C10—H10B	109.5
S1—C1—H1	106.9	C8—C10—H10C	109.5
O2—C2—C3	113.08 (17)	H10A—C10—H10C	109.5
O2—C2—C1	107.21 (18)	H10B—C10—H10C	109.5
C3—C2—C1	108.36 (17)	O6—C11—H11A	109.5
O2—C2—H2A	109.4	O6—C11—H11B	109.5
C3—C2—H2A	109.4	H11A—C11—H11B	109.5
C1—C2—H2A	109.4	O6—C11—H11C	109.5
O3—C3—C2	109.57 (17)	H11A—C11—H11C	109.5
O3—C3—C4	109.76 (16)	H11B—C11—H11C	109.5
C2—C3—C4	110.45 (17)	O7—C12—H12A	109.5
O3—C3—H3	109.0	O7—C12—H12B	109.5
C2—C3—H3	109.0	H12A—C12—H12B	109.5
C4—C3—H3	109.0	O7—C12—H12C	109.5
O4—C4—C5	108.48 (16)	H12A—C12—H12C	109.5
O4—C4—C3	109.26 (15)	H12B—C12—H12C	109.5
C5—C4—C3	109.28 (16)	C14—C13—C18	119.2 (2)
O4—C4—H4	109.9	C14—C13—S1	119.62 (17)
C5—C4—H4	109.9	C18—C13—S1	121.16 (18)
C3—C4—H4	109.9	C15—C14—C13	120.4 (2)
O1—C5—C6	107.47 (15)	C15—C14—H14	119.8
O1—C5—C4	107.16 (16)	C13—C14—H14	119.8
C6—C5—C4	114.53 (16)	C14—C15—C16	120.7 (2)
O1—C5—H5A	109.2	C14—C15—H15	119.6
C6—C5—H5A	109.2	C16—C15—H15	119.6
C4—C5—H5A	109.2	C17—C16—C15	118.3 (2)
O5—C6—C5	113.01 (18)	C17—C16—C19	120.8 (2)
O5—C6—H6A	109.0	C15—C16—C19	121.0 (2)
C5—C6—H6A	109.0	C18—C17—C16	121.3 (2)
O5—C6—H6B	109.0	C18—C17—H17	119.3
C5—C6—H6B	109.0	C16—C17—H17	119.3
H6A—C6—H6B	107.8	C17—C18—C13	120.1 (2)
O6—C7—O3	110.48 (16)	C17—C18—H18	120.0
O6—C7—C9	113.07 (18)	C13—C18—H18	120.0
O3—C7—C9	106.22 (16)	C16—C19—H19A	109.5
O6—C7—C8	103.52 (16)	C16—C19—H19B	109.5
O3—C7—C8	109.92 (16)	H19A—C19—H19B	109.5
C9—C7—C8	113.69 (17)	C16—C19—H19C	109.5
O7—C8—O4	110.16 (16)	H19A—C19—H19C	109.5
O7—C8—C10	113.29 (17)	H19B—C19—H19C	109.5
			
C5—O1—C1—C2	−55.7 (2)	C3—O3—C7—O6	58.6 (2)
C5—O1—C1—S1	69.57 (19)	C3—O3—C7—C9	−178.44 (16)
C13—S1—C1—O1	52.60 (16)	C3—O3—C7—C8	−55.0 (2)
C13—S1—C1—C2	179.90 (15)	C12—O7—C8—O4	56.1 (2)
O1—C1—C2—O2	−71.4 (2)	C12—O7—C8—C10	−61.6 (2)
S1—C1—C2—O2	161.43 (14)	C12—O7—C8—C7	175.43 (16)
O1—C1—C2—C3	50.9 (2)	C4—O4—C8—O7	60.73 (19)
S1—C1—C2—C3	−76.21 (18)	C4—O4—C8—C10	−176.74 (16)
C7—O3—C3—C2	−178.78 (17)	C4—O4—C8—C7	−54.5 (2)
C7—O3—C3—C4	59.8 (2)	O6—C7—C8—O7	174.85 (15)
O2—C2—C3—O3	−56.4 (2)	O3—C7—C8—O7	−67.12 (19)
C1—C2—C3—O3	−175.15 (17)	C9—C7—C8—O7	51.8 (2)
O2—C2—C3—C4	64.6 (2)	O6—C7—C8—O4	−66.44 (19)
C1—C2—C3—C4	−54.1 (2)	O3—C7—C8—O4	51.6 (2)
C8—O4—C4—C5	177.32 (16)	C9—C7—C8—O4	170.49 (16)
C8—O4—C4—C3	58.3 (2)	O6—C7—C8—C10	51.5 (2)
O3—C3—C4—O4	−59.4 (2)	O3—C7—C8—C10	169.51 (17)
C2—C3—C4—O4	179.71 (16)	C9—C7—C8—C10	−71.6 (2)
O3—C3—C4—C5	−177.92 (15)	C1—S1—C13—C14	−107.21 (19)
C2—C3—C4—C5	61.2 (2)	C1—S1—C13—C18	70.17 (19)
C1—O1—C5—C6	−177.40 (17)	C18—C13—C14—C15	0.3 (3)
C1—O1—C5—C4	59.0 (2)	S1—C13—C14—C15	177.77 (18)
O4—C4—C5—O1	−179.39 (14)	C13—C14—C15—C16	−1.2 (4)
C3—C4—C5—O1	−60.3 (2)	C14—C15—C16—C17	1.4 (3)
O4—C4—C5—C6	61.5 (2)	C14—C15—C16—C19	−177.1 (2)
C3—C4—C5—C6	−179.45 (17)	C15—C16—C17—C18	−0.8 (3)
O1—C5—C6—O5	−60.6 (2)	C19—C16—C17—C18	177.7 (2)
C4—C5—C6—O5	58.3 (2)	C16—C17—C18—C13	0.0 (3)
C11—O6—C7—O3	53.1 (2)	C14—C13—C18—C17	0.3 (3)
C11—O6—C7—C9	−65.8 (2)	S1—C13—C18—C17	−177.13 (17)
C11—O6—C7—C8	170.71 (17)		

### Hydrogen-bond geometry (Å, °)

**Table:**

*D*—H···*A*	*D*—H	H···*A*	*D*···*A*	*D*—H···*A*
O2—H2···O5^i^	0.86 (3)	1.96 (3)	2.788 (2)	161 (2)
O5—H5···O7^ii^	0.80 (2)	2.21 (3)	2.971 (2)	158 (3)

Symmetry codes: (i) −*x*+1, *y*+1/2, −*z*+1; (ii) −*x*+1, *y*−1/2, −*z*+1.
